# Unveiling the Reversibility and Stability Origin of the Aqueous V_2_O_5_–Zn Batteries with a ZnCl_2_ “Water‐in‐Salt” Electrolyte

**DOI:** 10.1002/advs.202102053

**Published:** 2021-10-19

**Authors:** Xiaoyu Tang, Pan Wang, Miao Bai, Zhiqiao Wang, Helin Wang, Min Zhang, Yue Ma

**Affiliations:** ^1^ State Key Laboratory of Solidification Processing Center for Nano Energy Materials School of Materials Science and Engineering Northwestern Polytechnical University and Shaanxi Joint Laboratory of Graphene (NPU) Xi'an 710072 China; ^2^ State Key Laboratory of Solidification Processing School of Materials Science and Engineering Northwestern Polytechnical University Xi'an 710072 China; ^3^ Training Center for Engineering Practices Northwestern Polytechnical University Xi'an 710129 China

**Keywords:** operando XRD, proton insertion mechanism, self‐discharge, V_2_O_5_ cathode, water‐in‐salt electrolytes

## Abstract

Aqueous V_2_O_5_–Zn batteries, an alternative chemistry format that is inherently safer to operate than lithium‐based batteries, illuminates the low‐cost deployment of the stationary energy storage devices. However, the cathode structure collapse caused by H_2_O co‐insertion in aqueous solution dramatically deteriorates the electrochemical performance and hampers the operation reliability of V_2_O_5_–Zn batteries. The real‐time phase tracking and the density functional theory (DFT) calculation prove the high energy barrier that inhibits the Zn^2+^ diffusion into the bulk V_2_O_5_, instead the ZnCl_2_ “water‐in‐salt electrolyte” (WiSE) can enable the dominant proton insertion with negligible lattice strain or particle fragment. Thus, ZnCl_2_ WiSE enables the enhanced reversibility and extended shelf life of the V_2_O_5_–Zn battery upon the high temperature storage. The improved electrochemical performance also benefits by the inhibition of vanadium cation dissolution, enlarged voltage window, as well as the suppression of the Zn dendrite protrusion. This study comprehensively elucidates the pivotal role of a concentrated ZnCl_2_ electrolyte to stabilize the aqueous batteries at both the static storage and dynamic operation scenarios.

## Graphical abstract

The application of ZnCl_2_ “water‐in‐salt” electrolyte in V_2_O_5_—Zn battery can effectively enhance the electrochemical performance no matter in dynamic or static conditions. This improvement is ascribed to the inhibition of cathode dissolution, extended voltage window, and the suppression of the Zn dendrite protrusion. Moreover, it is proved that proton inertion contributes most of the capacity, benefiting the particle integrity.

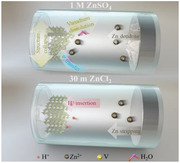

## 1 Introduction

With the uptake of intermittent renewable generation (namely wind and solar power), the grid‐scale energy storage and behind‐the‐meter storage markets rapidly grow with the more demanding requirements for the greener, cleaner, and more cost‐efficient chemistry formats.^[^ ^1^, ^2^, ^3^, ^4^ ^]^ The aqueous zinc batteries (AZBs) thus stand out as an ideal alternative to fire‐prone lithium batteries for stationary storage applications due to the scalability, operation reliability, and lower production cost.^[^ ^5^, ^6^, ^7^, ^8^, ^9^, ^10^ ^]^ Meanwhile, zinc metal possesses a low equilibrium redox potential (−0.76 V vs standard hydrogen electrode) and high hydrogen evolution overpotential, balancing the appropriate energy density with aqueous electrolyte compatibility. Despite these merits, the AZBs suffer from cathode structural collapse and electrolyte decomposition, especially at the slow cycling rates. Besides, the self‐discharge issue of AZBs has yet to be explored, posing the limited calendar life for the practical deployment of the AZBs.^[^ ^10^, ^11^ ^]^ V_2_O_5_ and its derivative are the most studied cathode materials for the AZBs because of the natural abundance and rich oxidation states of vanadium. Noteworthy, the charge charrier for V_2_O_5_ in typical AZBs is the hydrated Zn ions other than Zn ions, due to the high charge density of Zn ion and the charge shielding effect of water molecular.^[^ ^12^, ^13^, ^14^ ^]^ The water and Zn ion co‐insertion would cause dramatic lattice variation up to 300% and deteriorate the structural stability of the strained V_2_O_5_ upon the long‐term cycling.

Pioneer studies mainly focused on the nano‐engineering techniques, that is, the doping process or interfacial modification for structural stabilization.^[^ ^15^, ^16^, ^17^, ^18^ ^]^ Additionally, the cation/ molecular pre‐intercalation methods, including Li^+^, Na^+^, K^+^, Mg^2+^, Cu^2+^, Zn^2+^, Ni^2+^, Mn^2+^, H_2_O, and polyaniline, were proposed to buffer the lattice expansion of the layered galleries and alleviate the internal stress.^[^ ^19^, ^20^, ^21^, ^22^, ^23^, ^24^, ^25^, ^26^, ^27^, ^28^, ^29^, ^30^, ^31^, ^32^ ^]^ For instance, Yang et al. reported a cotton‐like Li^+^ pre‐intercalated V_2_O_5_ material, demonstrating an enlarged layered spacing (13.77 Å) and facile Zn^2+^ diffusion (up to 3.37 × 10^−8^ cm^2^ s^−1^).^[^ ^28^ ^]^ Liu and his co‐workers synthesized polyaniline intercalated V_2_O_5_ with constant interlayer distance upon the intercalation and extraction of the Zn^2+^.^[^ ^32^ ^]^ Even though the nano‐engineering and pre‐intercalation strategies achieved great success in terms of capacity and cycle stability at high rate, the complex pre‐treatment procedures hinder the practical application of the as‐synthesis materials, while the pre‐intercalation processing would also sacrifice the rated gravimetric/volumetric energy density of the AZBs. At the high voltage status, moreover, the large surface area of the nanostructure would cause serious interfacial side reactions upon the electrolyte contact. Thus, the innovative design of the practical AZB should put more emphasis on the component compatibility of the AZB system, besides the design ingenuity of the electrodes only.

The high concentration of the “water‐in‐salt” (WiSE) electrolyte was recently proposed to optimize the cyclability of AZBs. Wang and coworkers reported the 21 m LiTFSI (lithium bis(trifluoromethanesulfonyl)imide) salt could extend the electrochemical window of the aqueous electrolytes to ≈3.0 V with the aid of solid electrolyte interface (SEI) formation.^[^ ^33^ ^]^ A composite electrolyte comprising 20 m LiTFSI and 1 m Zn(TFSI)_2_ was also employed to stabilize the reversibility of Zn batteries (Coulombic efficiency (CE) of 99.5% for 200 cycles at 1 mA cm^−2^).^[^ ^34^ ^]^ However, the heavy use of costly salts goes against the cost benefits of the aqueous electrolyte. Recently, Zhang et.al. used a new type of WiSE based on the low‐cost ZnCl_2_ to suppress the zinc dendrites.^[^ ^35^ ^]^ Jin and co‐workers developed a low‐cost 19 m (17 m NaClO_4_ + 2 m NaOTF) WISE for aqueous sodium ion batteries, in which NaClO_4_ was mainly used to reduce water activity as well as a small amount of NaOTF was applied to assist formation of robust SEI, enabling a wide electrochemical stability window of 1.6–4.4 V (vs Na^+^/Na).^[^ ^36^ ^]^ Despite the enhanced CE values achieved in these concentrated electrolytes, there remains a paucity for the spontaneous interfacial instability or electrochemical‐driven redox reaction in the aqueous batteries. It is noteworthy that the influence of electrolyte concentration on reaction mechanism of V_2_O_5_ cathode in AZBs has not been studied even though the water plays a pivotal role in the charge storage process.

Herein, we systematically investigated the detrimental impact of the H_2_O/Zn^2+^ co‐insertion into the V_2_O_5_ cathode on the micro‐ and electrode level. Correspondingly, we employed a high concentration of ZnCl_2_ WiSE (30 m) to eliminate the water co‐insertion process. The real‐time phase tracking and DFT calculations of the V_2_O_5_ cathode validate the negligible lattice variation accompanied by H^+^ intercalation/de‐intercalation upon the cycling in the WiSE. Moreover, the prototype realizes robust capacity retention at the low current rate (9.5% capacity loss after 300 cycles at 50 mA g^−1^) as well as self‐discharge suppression (74.66% capacity retention after 300 h at 55 °C). Based on these multiscale structural and compositional characterizations, we propose a plausible deterioration model of the vanadium‐based cathode in the dilute electrolyte: severe internal lattice stress of the V_2_O_5_ cathode that derives from the aqueous solvent media would induce the particle pulverization and cation dissolution, which further exacerbates the parasitic interfacial reaction in turn. On the contrary, the ZnCl_2_ WiSE could significantly immobilize the free water and stabilize the interfacial electrochemistry in the AZB, at both the high‐temperature storage and dynamic operation scenarios.

## 2 Results and Discussion

**Figure 1:**
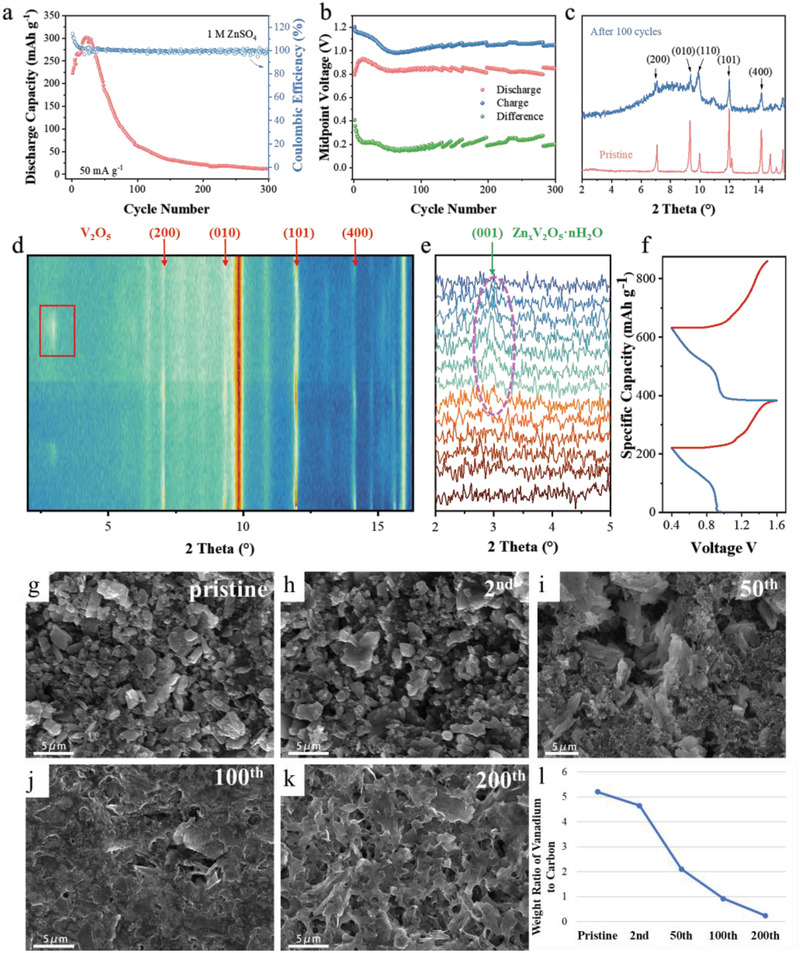
a) Cycling performance of the bulk V_2_O_5_ cathode in 1 m ZnSO_4_ at a current density of 50 mA g^−1^. b) The differential capacity curves during the cycling as shown in Figure 1a. c) XRD patterns obtained before cycle and after 100 cycles. d) The operando XRD pattern of the V_2_O_5_ cathode in 1 m ZnSO_4_ electrolyte for the initial two cycles. e) Enlarged view of the selected region in Figure 1d (marked by the red rectangle). f) The galvanostatic charge–discharge curves corresponding to the dynamic phase transition process as shown in Figure 1d. The morphology evolution of the V_2_O_5_ cathode g) before cycling and after h) 2, i) 50, j) 100, and k) 200 cycles. l) The corresponding weight ratio of vanadium to carbon detected by EDS.

As shown in Figure S1a, Supporting Information, the milled V_2_O_5_ particles are ≈4–5 µm in size with irregular interparticle pores distributed on the surface. In addition, the energy‐dispersive X‐ray spectroscopy (EDS) result of the top‐view electrode reveals the uniform dispersion of the graphite and V_2_O_5_ particles on the scale of micrometer. The as‐prepared V_2_O_5_ cathode was directly assembled as a reference to the zinc foil to illuminate the impact of various electrolytes on cycle performance. The commonly employed electrolyte (1 m ZnSO_4_) realized a maximum capacity of 302 mA h g^−1^ for AZBs (**Figure** **1**a). Noteworthy, the capacity of aqueous battery fades rapidly at the low current density of 50 mA g^−1^: only 4% original capacity was maintained after 300 cycles.^[^ ^35^ ^]^

To probe the origin of this fast capacity decay, electrochemical, structural, and compositional characterizations were conducted. First, the difference between charge/discharge midpoint voltage was ≈0.2–0.3 V after the first ten cycles, suggesting that the dramatic capacity loss of V_2_O_5_ in 1 m ZnSO_4_ was not caused by kinetic factors. In terms of active material structure, the X‐ray diffraction pattern of the as‐prepared cathode material exhibits the main peaks of additive graphite (PDF NO.89‐8487) and phase‐pure V_2_O_5_ (PDF NO.75‐457), demonstrating that the ball‐milling process does not induce any undesired phase transition (Figure 1c). After 100 cycles, the peak intensity decreased seriously, especially for the (010) peak, indicating the damaged crystal structure and the delamination phenomenon. Corresponding, the particle fracture, exfoliation of the layered crystal, and abundant defects on the surface have been observed after 50 cycles in 1 m ZnSO_4_ (Figure S2, Supporting Information).

To futher probe the relationship between dynamic phasic transition and the properties and structure change, as shown in Figure 1d–f, we conducted the operando XRD test for the V_2_O_5_ cathode in the dilute electrolyte (1 m ZnSO_4_). Obviously, a new peak that appeared at the discharged state was indexed to the (001) peak of Zn*_x_* V_2_O_5_·nH_2_O, suggesting the co‐intercalation of the Zn^2+^ as well as the accompanied water molecules insertion into the V_2_O_5_ structure.^[^ ^12^, ^13^, ^20^ ^]^ Pioneer works also reported that the water molecular would intercalate into the layered spacing of V_2_O_5_ and induce the dramatic lattice variation.^[^ ^12^, ^37^ ^]^ In fact, the V_2_O_5_ cathode in dilute electrolyte would experience a serious lattice swelling from 4.4 to 13.9 Å (319%). Post‐mortem morphological analysis was carried out to probe the electrode evolution upon such mechanical stress. Before cycling, the cathode displays randomly distributed V_2_O_5_ particles. Then, the particles gradually evolve into the interconnected porous structure in the dilute electrolyte after 200 cycles. Noteworthy, this microstructure evolution is accompanied by serious active material loss, evidenced by the gradually decreased V:C ratio from 5.20 for the pristine electrode to 0.25 for the cathode cycled in 1 m ZnSO_4_ (Figure 1l). In this regard, the active material loss that derived from the delamination and particle fragmentation, should be the main reason for the capacity fading at the microscale.

**Figure 2:**
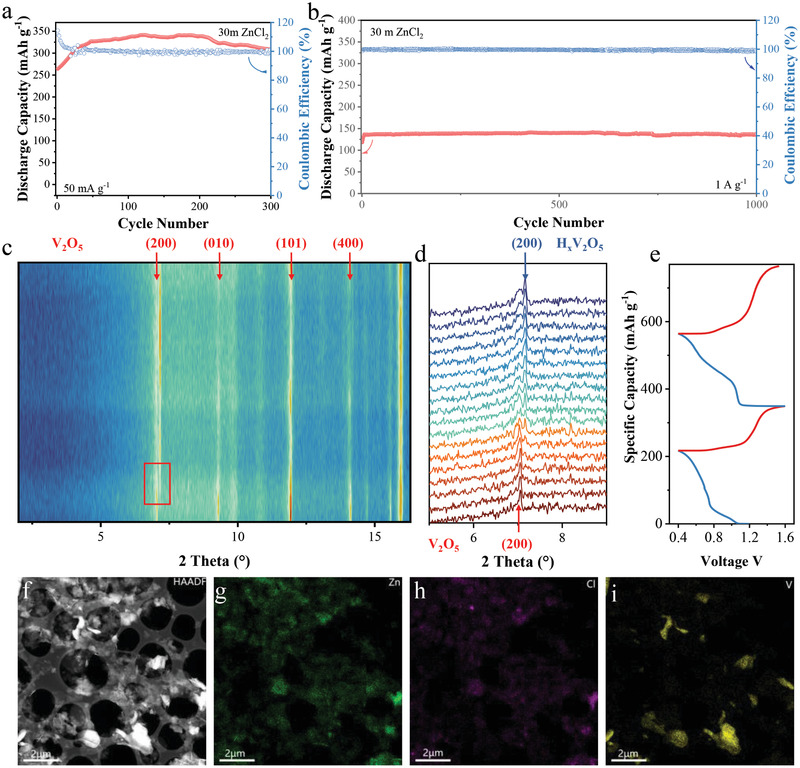
a) Cycling performance of the bulk V_2_O_5_ cathode in 30 m ZnCl_2_ at a current density of 50 mA g^−1^. b) Long‐cycling performance of V_2_O_5_ in WiSE at 1 A g^−1^.c) The operando XRD patterns of V_2_O_5_ in the 30 m ZnCl_2_ electrolytes for the initial two cycles. d) Enlarged view of the selected region in Figure 2c (marked by the red rectangle). e) The galvanostatic charge–discharge curves corresponding to the dynamic phase transition process as shown in Figure 2c. f) The HAADF image and corresponding energy dispersive X‐ray spectra with the elemental maps of g) Zn, h) Cl, and i) V obtained from the discharged V_2_O_5_ electrode cycled in WiSE.

From the experimental results of V_2_O_5_ cathode cycled in 1 m ZnSO_4_, the water co‐insertion damages the crystal structure seriously. We employed the high concentration electrolyte (30 m ZnCl_2_) to probe the relationship between electrochemical reaction and water content. The application of WiSE effectively improved the cycle stability of V_2_O_5_ cathode: the maximum capacity was evaluated 341.0 mA h g^−1^ and only 9.5% capacity decay can be observed after 300 cycles at 50 mA g^−1^ (**Figure** **2**a). The electrode experienced a gradual capacity increase, in accompaniment with the over 100% coulombic efficiency at the initial cycles. It indicates that the charge carriers that intercalated into the cathode lattice cannot be extracted completely and thus, more and more low‐valence‐state vanadium should exist in the cathode upon the activation process. Upon the initial activation process, the mixed valence state of the V_2_O_5_ improved the electron conductivity and accounted for an increased capacity.^[^ ^38^, ^39^, ^40^ ^]^ At the current density of 1 A g^−1^, the WiSE enables the ultra‐robust durability of AZBs with negligible capacity decay for 1000 cycles.

According to the operando XRD test, the (200) peak of V_2_O_5_ cathode shows a typical two‐phase transition behavior upon the discharge process in the WiSE: the intensity of the peak located at ≈7.05° gradually decreases and an approximate peak appears at ≈7.17°, indicating that a new crystal phase transformed from the pristine V_2_O_5_ emerges. Besides, the diffraction peak indexed to the Zn intercalated vanadium oxide or hydrated vanadium oxide cannot be observed. To further illuminate the abnormal lattice variation, Figure 2f–i presents the scanning transmission electron microscopy (STEM) and the corresponding EDS images of the discharged cathode. The elemental map of V differs from the Zn map while the dispersion of Zn and Cl elements are imposable. Combining with the operando XRD results, we thus speculate that the H^+^ would continuously intercalate into the layered spacing to form H*_x_* V_2_O_5_ phase during the discharge process in the 30 m ZnCl_2_ electrolyte, while the increasing amount of OH^−^ leads to the formation of zinc hydroxide chloride corresponding to the Zn/Cl rich region in the EDS results. A similar H^+^ insertion phenomenon has been previously reported for the MnO_2_ cathodes.^[^ ^41^, ^42^ ^]^

**Figure 3:**
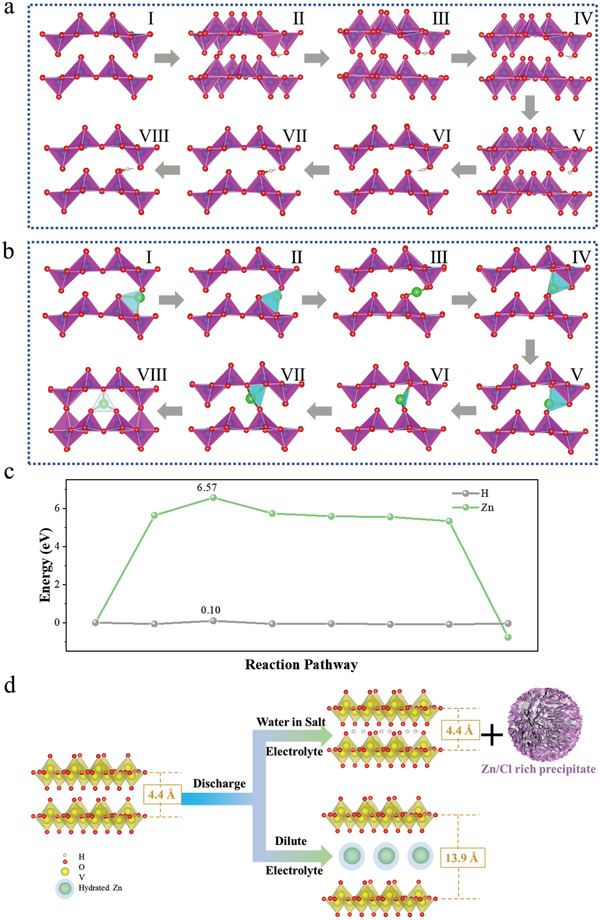
a) H^+^ and b) Zn^2+^ migration pathway and its c) corresponding energy barriers. d) Schematic illustration of the dynamic structural evolution for V_2_O_5_ cathode in different electrolytes.

To fully understand the competitive relationship between the charge carriers, the dynamic kinetics of H^+^ and Zn^2+^ diffusion in V_2_O_5_ were also calculated by simulating the structural evolution in the absence of water. The migration pathways for H^+^ and Zn^2+^ in V_2_O_5_ are shown in **Figure** **3**a,b while the corresponding energy barriers are plotted in Figure 3c. The diffusion barriers of H^+^ and Zn^2+^ in V_2_O_5_ are 0.10 and 6.57 eV. The diffusion barrier of V_2_O_5_ can even block the Zn^2+^ intercalation in WiSE. According to the aforementioned experimental results, Figure 3d schematically illustrates that the V_2_O_5_ cathode in dilute electrolyte would experience a serious lattice swelling from 4.4 to 13.9 Å (319%) while the application of WiSE effectively suppresses the lattice expansion due to the small radius of the proton. Based on the plausible proton insertion mechanism, the large volume change and high internal stress of the V_2_O_5_ cathode could be remarkably avoided in the WiSE.

**Figure 4:**
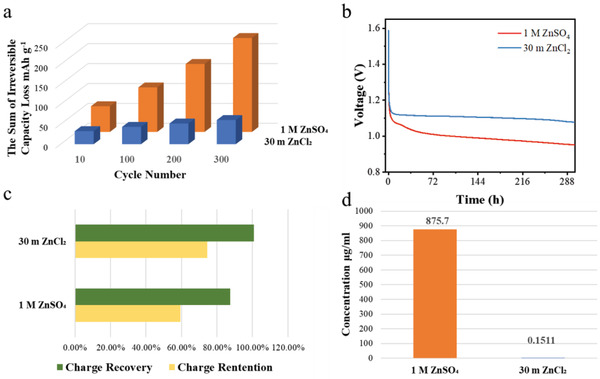
a) The sum of irreversible capacity loss (ICL) during the cycling at 50 mA g^−1^. b) The open circuit voltage (OCV) profiles of V_2_O_5_ cathode at 55 °C in various electrolytes. c) The charge retention and charge recovery of the V_2_O_5_ cathode after the high temperature storage (55 °C) in various electrolytes. d) The concentration of the V element in the electrolytes with V_2_O_5_ powder staying for 10 days at 55 °C.

Besides the cycle performance and bulk structure evolution of cathode, we also evaluated the compatibility of V_2_O_5_ cathode and electrolyte in detail. As shown in **Figure** **4**a, the irreversible capacity loss (ICL) for the model with the WiSE accumulated to 60.6 mA h g^−1^ upon 300 cycles, while the cells containing 1 m ZnSO_4_ piled up the irreversible capacity consumption to 235.6 mA h g^−1^, consistentwith the excellent cycle stability. The self‐discharge rate is a critical indicator to assess the rechargeable battery in the practical application scenarios, especially upon the operation at elevated temperatures. Therefore, the above various cells were charged to 1.6 V and kept idling at 55 °C to evaluate the self‐discharge rate. Subsequently, all the cells were discharged and charged again at 20 mA g^−1^. As compared in Figure 4b, the cathode in the WiSE displays the highest OCV value of 1.08 V after the storage process. Correspondingly, the cell with the high concentration electrolytes exhibits the enhanced levels of the capacity retention and charge recovery: only 25.54% capacity decayed upon the idling state and almost all the Zn^2+^ could be extracted in the subsequent charge process, suggesting that reversible side reactions had been reduced and no irreversible structural damage occurred for the cathode.

Additionally, the capability of various electrolytes against oxidation reaction at high voltage was measured by linear sweep voltammetry (LSV) with titanium foil as the working electrode in three‐electrode cells at a scan rate of 0.1 mV s^−1^ (Figure S8, Supporting Information). From 1 to 30 m, the oxidative potential of the ZnCl_2_ solutions is increased from 2.10 to 2.54 V versus Zn/Zn^2+^, suggesting the enlarged voltage window. The increasing trend of electrochemical stability echoes with the capacity retention/recovery rate as shown in Figure 4c. To exclude the influence of kinetic factor during the linear sweep voltammetry test, we coated activated carbon on the Ti foil and paired it with 30 m ZnCl_2_ electrolyte and Zn foil, then charged it with a constant current (0.1 mA). We believe that the large surface area and good conductivity of activated carbon can reduce the electrolyte oxidation, and we find that the Cl_2_ evolution occurred above 2.0 V versus Zn/Zn^2+^. Thus, it should be no Cl_2_ evolution considering our onset potential is only 1.6 V versus Zn/Zn^2+^.

The concentrated electrolyte was proposed to suppress the metal ion dissolution of the layered cathode.^[^ ^35^ ^]^ Herein, the AZBs were shelved in various electrolytes at 55 °C for 10 days. Figure 4d; Figure S6, Supporting Information demonstrate the severe vanadium dissolution of the cathode in the 1 m ZnSO_4_ electrolyte, which subsequently induced the rapid capacity loss both upon the galvanostatic cycling and the high temperature storage. In sharp contrast, the concentrated ZnCl_2_ electrolyte would inhibit the vanadium dissolution due to the fewer free solvent molecules that can coordinate with the metal cations.^[^ ^43^, ^44^, ^45^ ^]^ In this regard, the usage of WiSE indeed practically obstructs the unwanted side reactions on the cathode/electrolyte interface both at the static and dynamic conditions.

**Figure 5:**
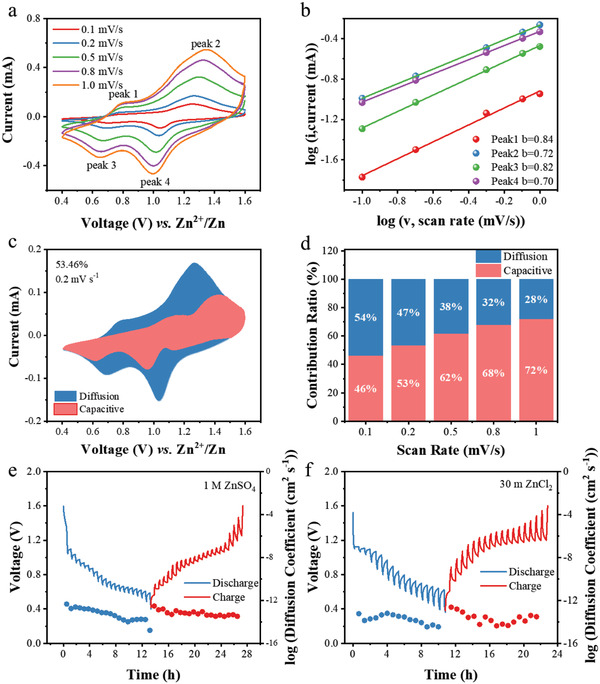
a) CV curves of the V_2_O_5_ cathode in ZnCl_2_ WiSE at different scan rates. b) Log *i* versus log *v* plots based on the CV profiles at the different oxidation/reduction states. c) Capacitive contribution current at 0.2 mV s^−1^. d) The capacitive contribution ratio at various sweep rates. Charge–discharge GITT curves at a current density of 60 mA g^−1^ and the corresponding ion diffusion coefficients in e) 1 m ZnSO_4_ and f) 30 m ZnCl_2_.

To further understand the energy storage behavior of V_2_O_5_ cathode in WiSE, cyclic voltammetry (CV) curves at different scan rates were tested to quantitatively identify the capacitive contribution as shown in **Figure** **5**. Two couples of redox peaks in CV curves remain the same with profiles obtained from the battery with dilute electrolyte (1 m ZnSO_4_).^[^ ^15^ ^]^ Upon the scan rate increase, the voltage hysteresis between the reduction peaks and the oxidation peaks gradually enlarged. According to previous reports, the capacitive effect of the battery system can be calculated by the relation *i* = *av^b^*. If *b* reaches 0.5, a faradic intercalation controls the charge storage process while *b* equals 1.0 for the pseudo‐capacitance process. The *b* values are calculated as 0.84, 0.72, 0.82, and 0.70 for peaks 1–4, respectively, suggesting the capacity of V_2_O_5_ particles is coherently dominated by the capacitive and diffusion behaviors. At a scan rate of 0.2 mV s^−1^, capacitive behavior contributes 53.46% of the total capacity and the capacitive contribution increases with scan rate. Therefore, the capacitance is a major contribution to the total capacity, especially at high charge/discharge rates. The galvanostatic intermittent titration technique (GITT) was also employed to investigate the ion diffusion rate. Despite the multivalence of Zn^2+^, the Zn^2+^ diffusion coefficient (≈10^−13^ cm^2^ s^−1^) in 1 m ZnSO_4_ is measured as one order magnitude higher than the H^+^ diffusion coefficient in WiSE (≈10^−14^ cm^2^ s^−1^). We ascribe this lower ion diffusion rate to the low charge charrier (H^+^) concentration in the WiSE, which in return guarantees the structural robustness of the V_2_O_5_ cathode at the low current density.

The dendrite formation would also deteriorate the cycle stability and CE, thus Zn dendrite inhibition is another urgent issue to be solved.^[^ ^46^, ^47^ ^]^ After 200 cycles, the zinc foil in 1 m ZnSO_4_ electrolyte shows the micro‐slice morphology due to the uncontrolled zinc plating (Figure S14, Supporting Information). In the dilute ZnCl_2_ electrolyte, similarly, the zinc nanoflakes with sharp edges randomly disperse on the Zn foil. As the concentration increases to 10 m, the number of nanoflakes decreases; while the WiSE electrolyte displays a fluffy morphology with smooth and dense surface morphology, without any dendrite or mossy zinc formation. The discharge/charge curves of a Zn/Zn symmetrical cell in different electrolytes are shown in Figure S15, Supporting Information. Compared with the apparent fluctuation of the voltage profile of 1 m ZnSO_4_, the 30 m ZnCl_2_ electrolyte shows more stability during Zn plating/stripping. These data strongly suggest that the ZnCl_2_ WiSE can effectively suppress the dendritic growth and enhance the cycling stability of zinc batteries.

**Figure 6:**
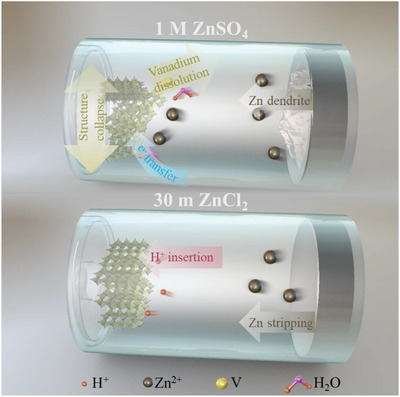
Schematic illustration of the electrochemical reaction for AZBs with 1 m ZnSO_4_ and 30 m ZnCl_2_ electrolytes.

The electrochemical reactions that occurred inside the V_2_O_5_–Zn batteries have been schematically illuminated in **Figure** **6**. In the dilute electrolyte of 1 m ZnSO_4_, the cathode would experience the structural collapse induced by the hydrated Zn ion intercalation, vanadium dissolution, and the electron transfer from the electrolyte. Moreover, the dendrite Zn formation would cause a serious safety concern. In comparison, the application of WiSE effectively suppresses the cathode loss, self‐discharge, and zinc dendrite growth. Noteworthy, the proton insertion charge storage process, instead of the hydrated Zn with large ion radius, prevents the layered structure delamination of cathode in WiSE. In fact, the concentrated ZnCl_2_ solution has been successfully used in the commercial “Zinc‐chloride” battery, with the similar proton insertion into the MnO_2_ lattice to provide capacity.^[^ ^48^ ^]^ However, the deeply discharged product, layered Mn(OH)_2_, cannot convert to the original MnO_2_ with tunnel structure. Thus, we believe that the V_2_O_5_–Zn battery with WiSE, which shows continuous layered structure change in cathode, is an alternative choice to be a large‐scale, low‐cost, and rechargeable energy supply.

## 3 Conclusion

In conclusion, we probed the reversibility and stability origin of the AZBs with the ZnCl_2_ WiSE through the real‐time phase tracking, both at the dynamic cycling and static storage scenarios. The experimental and theoretical analysis validated the enhanced mechanism of WiSE, namely the charge storage of the V_2_O_5_ cathode based on the H^+^ insertion/extraction process, which significantly inhibited the internal mechanical stress of the cathode structure as well as the Zn dendrite deposition. The interfacial stabilization of the AZB configuration thus echoes with the impressive cyclability (300 cycles at a low current density of 50 mA g^−1^) and the mitigated self‐discharge rate (74.66% capacity maintained after 300 h rest at the 55 °C). This work systematically provides a new insight into the AZBs integration, namely the component compatibility of the ZnCl_2_ WiSE, vanadium‐based cathode and the Zn foil. Additionally, a long‐standing puzzle for the structural evolution of the V_2_O_5_ cathode is elucidated, which highlights the crucial ZnCl_2_ WiSE in designing the highly efficient AZBs devices, both at the static storage and dynamic cycling conditions.

## 4 Experimental Section

### Materials Synthesis

All the chemicals were purchased from Sigma–Aldrich and used as received without further purification. V_2_O_5_ cathode material was prepared by a facile ball‐milling process. Briefly, V_2_O_5_ (99%) and graphite (99%) were mixed under ball‐milling with a predetermined weight ratio of 4:1 for 2 h at a speed of 250 r min^−1^. The ZnCl_2_ is easy to absorb the moisture in air, so the chemical should be stored and weighted in glove box filled with Ar. 30 m ZnCl_2_ aqueous solutions were prepared by dissolving 20.44 g zinc salts in 5 mL water solvent at 60 °C under stirring for 12 h. The as‐prepared samples also needed to be sealed for storage.

### Materials Characterization

Operando XRD analysis was carried out to study the phase transition during the discharge/charge process; the tests were performed in a transmission mode X‐ray diffractometer (STADIP STOE) with a position‐sensitive detector and Mo K*α*1 radiation with the wavelength (*λ*) of 0.70930 Å, operating at 50 kV and 40 mA. The crystal structure and lattice parameters of the obtained powder were analyzed by the same equipment. The particle size and morphology of the samples were examined by field emission scanning electron microscopy (Quanta 600 FEG) at 15 kV. Transmission electron microscopy (TEM) images were captured at 200 kV on an FEI Talos F200X with energy‐dispersive X‐ray spectroscopy (EDS). The compositional ratio of *V* in the cycled electrolyte was analyzed by inductively coupled plasma (ICP) analysis, which was performed on Agilent 5110.

### Electrochemical Measurements

The cathode slurry was prepared by mixing the active materials (70%), acetylene black (20%), and polyvinylidene fluoride (PVDF) binder (10%) in *N*‐methyl‐2‐pyrrolidone (NMP). Then, the homogenous slurry was spread onto a titanium foil and dried at 120 °C for 12 h in a vacuum oven. The final product was punched into discs (*Φ* = 12 mm) as the working electrode. The electrochemical performance was tested via a pouch cell where the zinc foil and Whatman GF/D glass fiber were used as a counter electrode and separator, respectively. The galvanostatic charge/discharge measurements were implemented on a Neware‐Battery Testing System 4 battery tester at ambient temperature within the potential range of 0.4–1.6 V. Linear sweep voltammetry (LSV) and cyclic voltammetry (CV) tests were carried out on a Gamry interface 1000 electrochemical workstation. LSV of electrolytes were also measured using a on a three‐electrode device at a scan rate of 0.1 mV s^−1^; the work electrode and counter electrode were both Ti foil, and Ag/AgCl electrode worked as the reference electrode. Ionic conductivity was measured at room temperature (298 K) by electrochemical impedance spectroscopy (EIS) with Gamry interface 1000 electrochemical workstation. The galvanostatic intermittent titration technique (GITT) measurements of the electrodes were conducted using a Neware‐Battery Testing System 4 battery tester at room temperature after initial three cycles. The test procedure was as follows: a 10 min galvanostatic discharge pulse (20 mA g^−1^) was applied to the cells, followed by 30 min of relaxation time without any current being passed through the cell. The cycle, consisting of a charge pulse and a relaxation period, was applied to the cell until its voltage reached 0.4 V versus Zn/Zn^2+^. Then, a charge pulse (20 mA g^−1^, 10 min) was applied to the cells, followed by a 30 min relaxation period without any current flowing through the cell. The cycle was applied to the cells until its voltage increased to 1.6 V versus Zn/Zn^2+^.

The chemical diffusion coefficient can be obtained as:

(1) $$D_{Zn^{2+}}=\frac{4}{\pi }{\left(\frac{n_{m}V_{m}}{S}\right)}^{2}{\left(\frac{\Delta E_{s}}{\Delta E_{\tau }}\right)}^{2}$$

Where *τ* is the constant current pulse duration (10 min); *n*_m_ and *V*_m_ are the moles (mol) of V_2_O_5_ and molar volume (cm^3^ mol^−1^), respectively; S is the electrode–electrolyte interface area (cm^−2^, taken as the geometric area of the electrode); *∆E*s and *∆Eτ* are the change in the steady state voltage and overall cell voltage after the application of a current pulse in a single step GITT experiment, eliminating the iR drop, respectively.

### Computational Details

All calculations were performed using Perdew–Burke–Ernzerhof (PBE) generalized gradient approximation (GGA) exchange–correlation implemented in the Vienna ab initio simulation package (VASP).^[^ ^49^, ^50^, ^51^ ^]^ The 2 × 1 × 1 supercell of V_2_O_5_ (010) with space group Pmm was used. The cutoff energy for all calculations was 400 eV. The Brillouin zone integration was accomplished with 2 × 2 × 8 Monkhorst–Pack k‐point mesh. The structures were relaxed until the energy difference and the residual force on the atoms reached 1 × 10^−4^ and 0.05 eV Å^−1^. During the optimization of the structures, the DFT + U with *U* = 3.25eV was considered.^[^ ^52^ ^]^ DFT‐D2 was used for the van der Waals' interactions between layers.^[^ ^53^ ^]^ To model ionic diffusion in the selected host structure, the climbing‐image nudged elastic band (CI‐NEB) method was employed to couple with density functional theory (DFT).^[^ ^54^ ^]^ For each CI‐NEB calculation, six images were interpolated between the initial and final structures. DFT + U was not used for the simulation of diffusion.^[^ ^55^, ^56^ ^]^ The convergence threshold of the total energy was set to 1 × 10^−4^ eV, and a tolerance of 0.1 eV Å^−1^ for the forces used in the CI‐NEB procedure.

## Conflict of Interest

The authors declare no conflict of interest.

## Supporting information

Supporting Information

## Data Availability

The data that support the findings of this study are available from the corresponding author upon reasonable request.
