# Supplementary material for: Unveiling the Reversibility and Stability Origin of the Aqueous V2O5–Zn Batteries with a ZnCl2 “Water‐in‐Salt” Electrolyte
Source: Adv Sci (Weinh). 2021 Oct 19;8(23):2102053. doi: 10.1002/advs.202102053 (PMC8655202; doi:10.1002/advs.202102053)
Supplement: Supplementary file 1 — Supporting Information [file ADVS-8-2102053-s001.pdf]

WILEY-VCH

© 2021 Wiley-VCH GmbH

ADVANCED SCIENCE
Open Access

Supporting Information

for *Adv. Sci.*, DOI: 10.1002/advs.202102053

Unveiling the Reversibility and Stability Origin of the Aqueous V_2O_5-Zn Batteries with a $ZnCl_2$ “Water-in-Salt” Electrolyte

*Xiaoyu Tang, Pan Wang, Miao Bai, Zhiqiao Wang, Helin Wang, Min Zhang and Yue Ma **

Supporting Information

Unveiling the Reversibility and Stability Origin of the Aqueous V_2O_5-Zn Batteries with a $ZnCl_2$ "Water-in-Salt" Electrolyte

*Xiaoyu Tang, Pan Wang, Miao Bai, Zhiqiao Wang, Helin Wang, Min Zhang and Yue Ma **

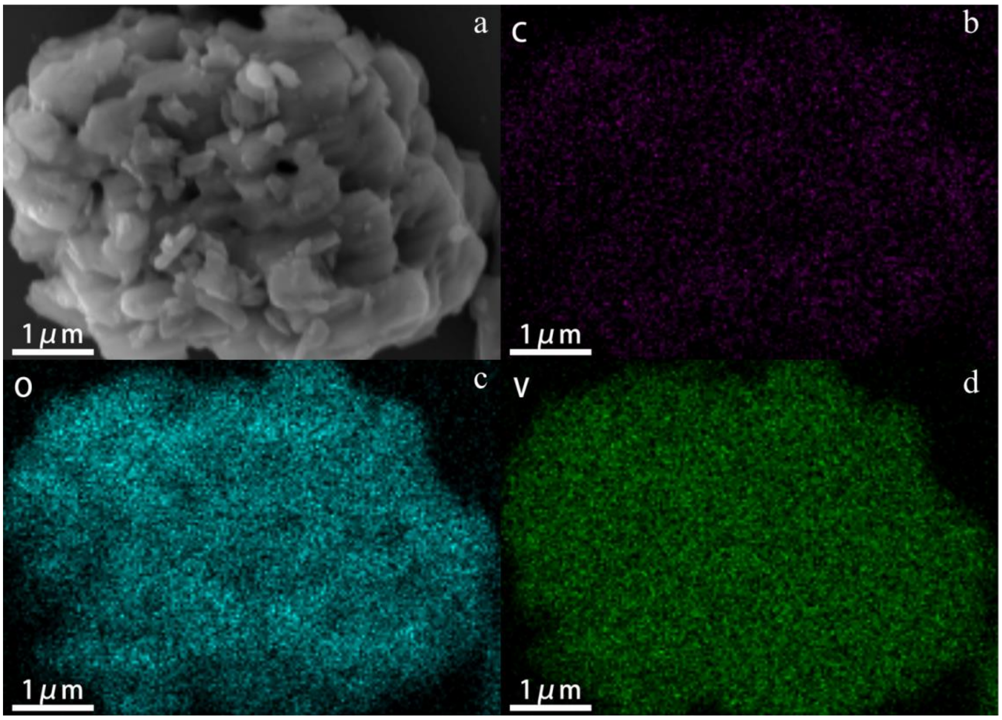

Figure S1 a) SEM image of the milled V_2O_5 cathode particle. b-d) The elemental maps of C, O, and V corresponding to Figure S1a.

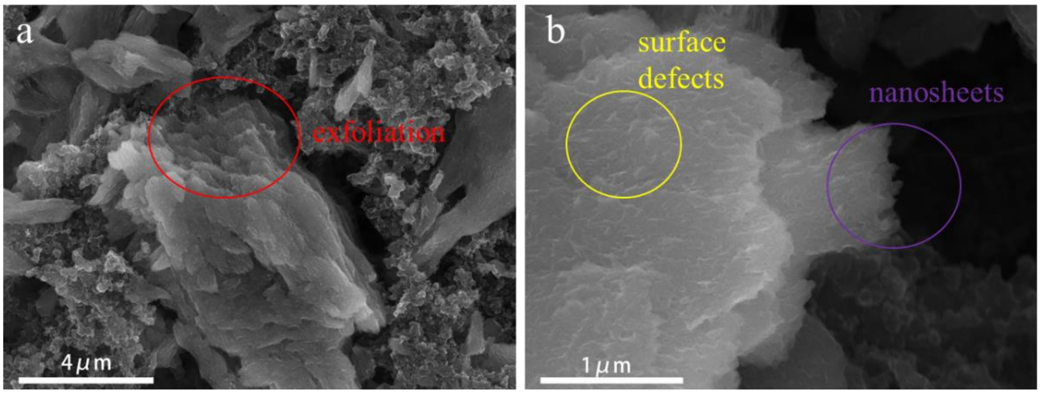

Figure S2 The post-mortem morphology of the V_2O_5 electrode after 50 cycles in 1 M $ZnSO_4$ at a) a low magnification and b) a higher magnification.

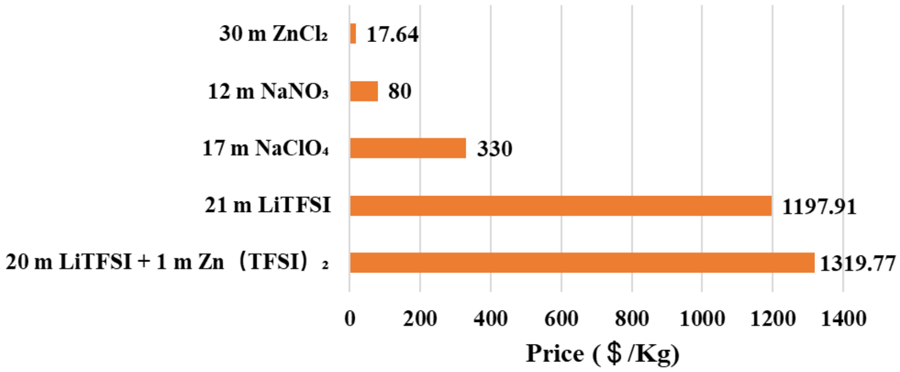

Figure S3 Price comparison of various “water-in-salt” electrolytes.

We have elaborated the characterizations of discharge product of V_2O_5 in 30 m $ZnCl_2$. The new peak in Figure 2 can be indexed to the (002) peak of $H_xV_2O_5$. To give more convincing evidence, we supplied the ex-situ XRD result of the V_2O_5 electrode at fully discharge state after 5 cycles in 30 m $ZnCl_2$. It displays that the all the peaks of the discharge product can be indexed to the $H_xV_2O_5$ phase. Combine with the electrochemical analysis and TEM result, the Zn ion does not insert into V_2O_5 cathode but precipitate on the cathode electrode. The analogous phenomenon is also reported for the MnO_2 cathode due to the H^+ insertion. Therefore, the discharge product should be assignable to the $H_xV_2O_5$.

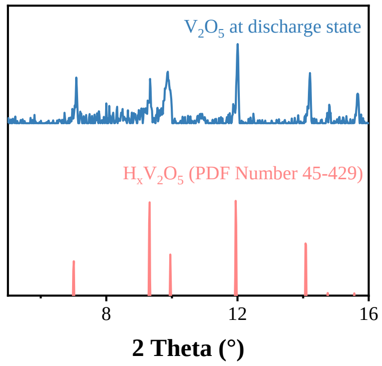

Figure S4 The ex-situ XRD pattern of the V_2O_5 cathode at the 6th discharge state in 30 m $ZnCl_2$.

To further probe the charge storage mechanism during the dynamic process, the Zn-V_2O_5 cells with WiSE electrolyte were cycled three times and disassembled at specific voltages (0.4, 0.6, 0.8, 1.0, 1.2 V) upon fourth discharge process, as shown in the Figure S5. According to the reviewer's suggestion, ICP tests were conducted to obtain the atomic ratio of Zn to V in the cathode at the different voltage. According to the Faraday formula, 1 mol electron transfer would cause 147.4 mAh g^{-1} capacity. The ratio of Zn to the transferred electron aroused from the electrochemical reaction was calculated by discharge capacity and the Zn/V ratio. As for the zinc intercalation reaction, 1 mol

zinc atom corresponds to 2 mol electron transfer. As shown in Figure S5a, the Zn/e- value is about 0.5 for 1 M $ZnSO_4$ electrolyte, suggesting the energy storage process is dominated by zinc intercalation reaction. Besides, the zinc intercalated into the V_2O_5 lattice would cause a gradually increased Zn/V value. As for the proton insertion mechanism, the precipitate can be depicted as $Zn_x(OH)_{2x}\cdot Zn_yCl_{2y}$. The Zn/e- value should be higher than 0.5 considering that the amount of OH^- should equal the transferred electron. The results show that the Zn/e^- value is about 0.61 at different voltages for WiSE. Thus, the proton insertion reaction runs through the entire energy storage process.

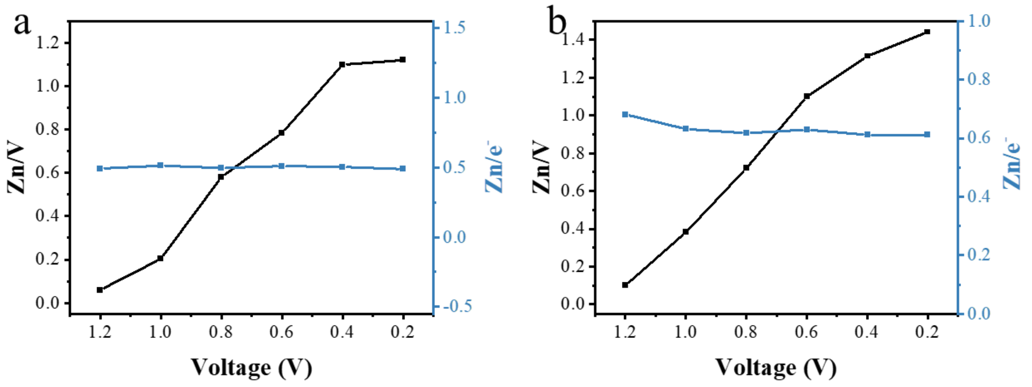

Figure S5 The atomic ratio of Zn/V and the calculated ratio of Zn to transferred electron obtained from V_2O_5 electrode cycled in a) 1 M $ZnSO_4$ and b) 30 m $ZnCl_2$ at different potentials.

The X-ray photoelectron spectroscopy (XPS) test for the V_2O_5 cathode cycled in WiSE at the discharged state was performed. The existence of Zn and Cl elements on the electrode surface indicates the formation of zinc hydroxide chloride deposits other than $ZnCl_2$ salt considering the electrode has been washed with deionized water before test. Additionally, the deconvolution of core level spectrum of V $2p_{3/2}$ confirms the electrochemical reduction of V−O−V upon discharge. The O 1s peak is split into two components: the peak at 530.2 eV refers to the V-O in the oxide species, while the peak at 531.3 eV originates from the OH^- in zinc hydroxide chloride, validating the formation of

the zinc hydroxide chloride species. The above analysis about XPS is consistent with the operando XRD and HRTEM results, evidencing the proton insertion mechanism.

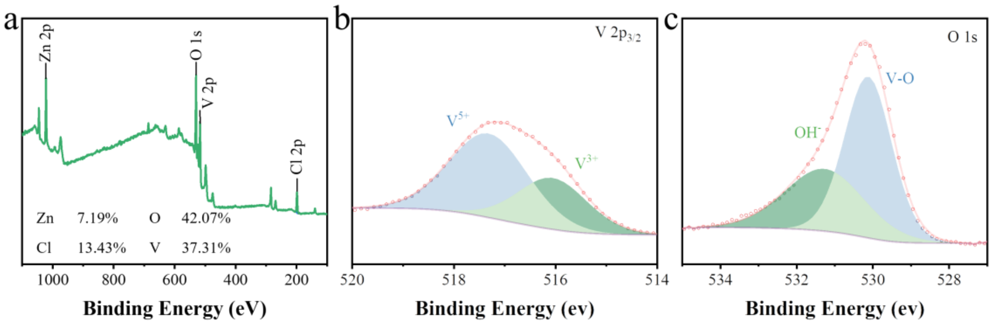

Figure S6 a) Wide-scan XPS spectra of the V_2O_5 cathode cycled in WiSE at discharged state. Detailed XPS spectra of b) V $2p_{3/2}$ and c) O 1s regions of the nanoflakes

We do not find the peak that indexed to the zinc hydroxide chloride species no matter in the *operando* XRD or *ex-situ* XRD spectra. HRTEM was conducted to investigate the more specific information of the zinc hydroxide chloride species. As shown in Figure S7, the Zn and Cl elements overlapped and covered on the V_2O_5, suggesting the OH^- induced zinc hydroxide chloride precipitate. We have selected a specific region marked by red rectangle, in which only Zn and Cl elements exist. The Figure S6b, corresponding to the zinc hydroxide chloride deposits, demonstrated no lattice fringe and the corresponding fast Fourier transform (FFT) pattern only displayed ambiguous rings. Thus, we think that no signal of zinc hydroxide chloride species can be observed in XRD test due to its relative amorphous nature.

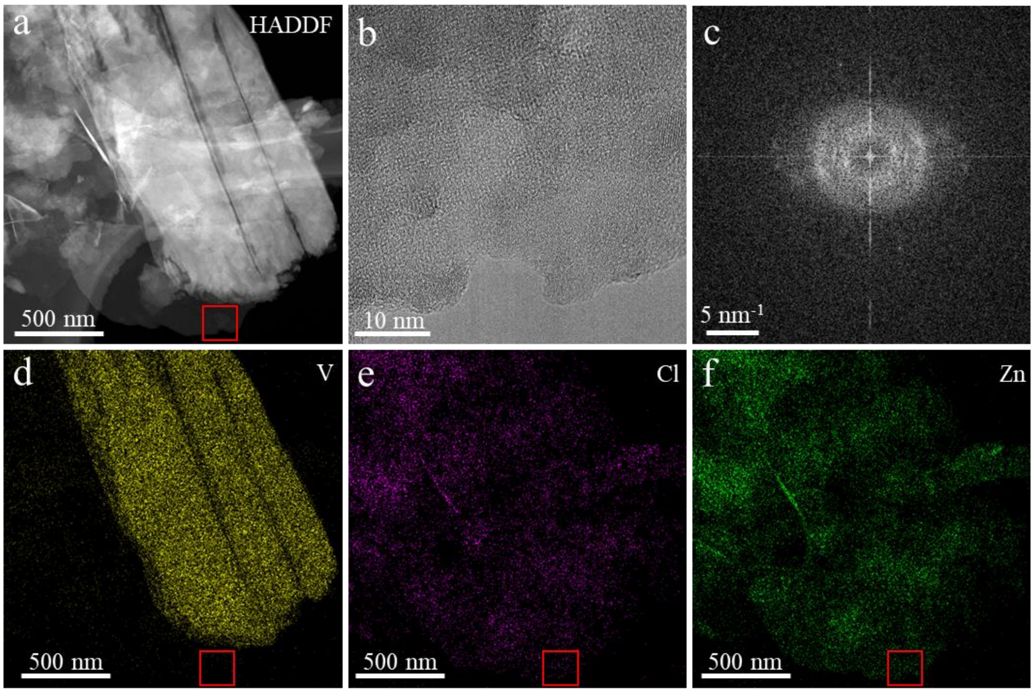

Figure S7 a) The HAADF image of the discharged V_2O_5 electrode cycled in WiSE. b) The HRTEM image of the selected region marked by red rectangle in Figure S7a. c) The FFT pattern obtained in Figure S7b. The energy dispersive X-ray spectra corresponding to Figure S7a with the elemental maps of d) V, e) Cl, and f) Zn.

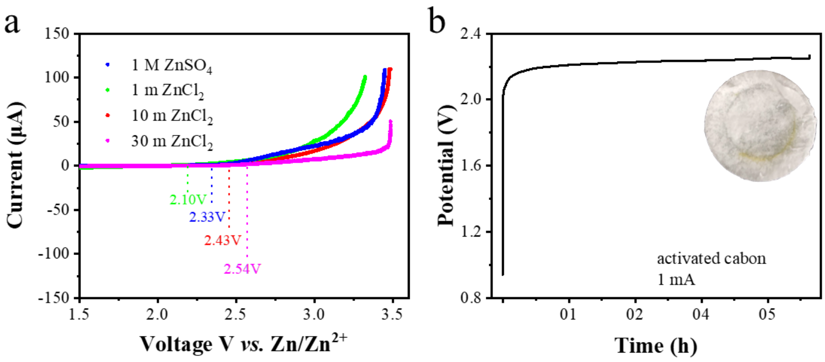

Figure S8 a) The electrochemical stability of various electrolytes evaluated by linear sweep voltammetry. b) The charge curve of the activated carbon electrode paired with 30 m $ZnCl_2$ electrolyte and Zn metal anode. Inset figure is the glass fiber separator which faces to the activated carbon working electrode.

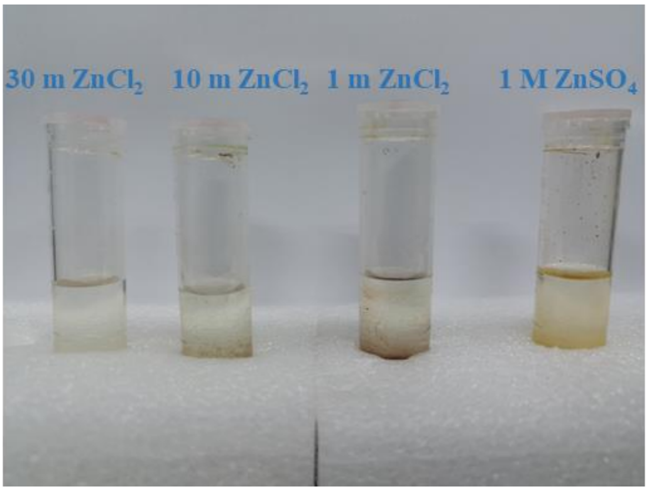

Figure S9 Optical images of various electrolytes with V_2O_5 powder staying for 10 days at 55 ºC.

The capacity loss caused by vanadium dissolving was further discussed. As shown in Figure 4d, there are 0.5 g V_2O_5 material in 5 mL electrolytes. The concentration of dissolved vanadium is 875.7 μg/ml. In other words, only 1.56% active material dissolved into the dilute electrolyte. We ascribe this large difference of vanadium dissolving amount in dynamic and static situations to the pulverization of the cathode particles upon cycling. Thus, the vanadium dissolution is the main reason of capacity decay, but it is also associated with the water co-intercalation process. To verify our hypothesis, the morphological evolution and statistical summary of the particle size distribution before and after static storage, corresponding to Figure S10 were evaluated. It can be observed that the V_2O_5 particle maintained the original morphology upon the storage. And the average particle size was about 5 μm before and after storage, demonstrating no particle pulverization within dilute electrolyte. Therefore, the water-insertion induced particle fracture and the subsequent vanadium dissolution should be the main reason of the capacity loss in dilute electrolyte.

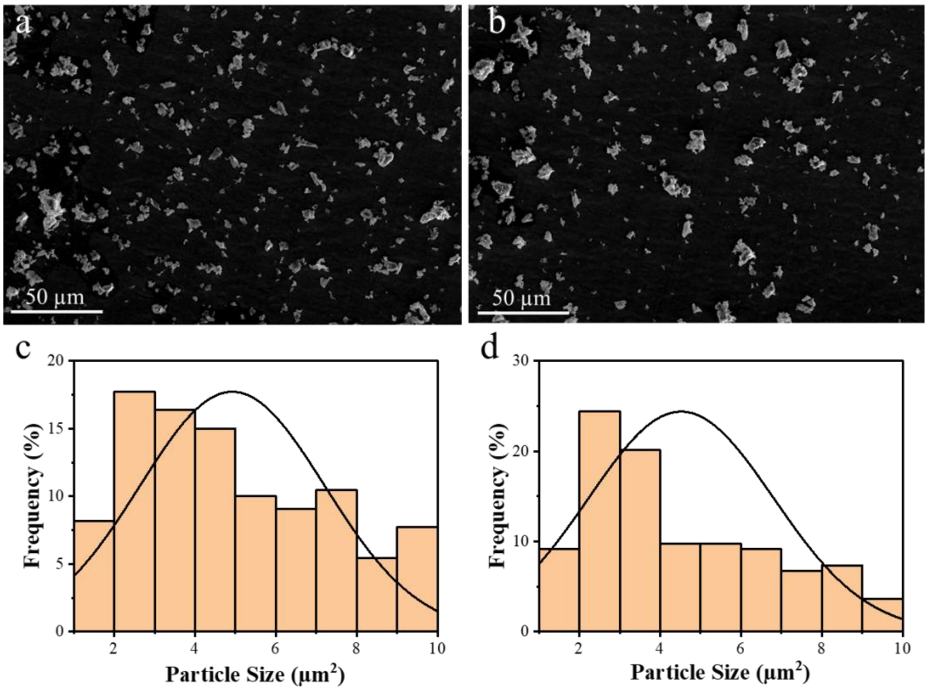

Figure S10 a) SEM images and c) corresponding histogram plots of V_2O_5 powder. b) SEM images and d) corresponding histogram plots of V_2O_5 powder staying in 1 M $ZnSO_4$ for 10 days at 55 ºC.

The decent kinetics in different electrolytes were further confirmed by electrochemical impedance spectroscopy (EIS) measurements. As shown in Figure S11 and Table S1, the V_2O_5 electrode cycled in 1 M $ZnSO_4$ displays very high charge-transfer resistance (R_{ct}) than electrode in 30 m $ZnCl_2$ before and after cycling. It is worth noting that there is an obvious decrease in the R_{ct} for the V_2O_5 in 1 M $ZnSO_4$ after ten cycles, which could be ascribed to the increased surface area due to particle fracture. Additionally, the electrode in 1 M $ZnSO_4$ has a faster ionic diffusion in the electrochemical processes, which is in agreement with the GITT results.

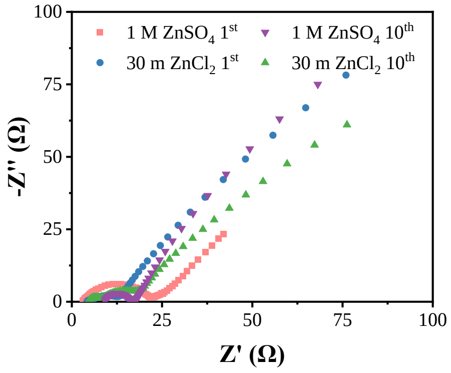

Figure S11 EIS spectra of V_2O_5 electrode in different electrolytes.

As shown in Figure S12, the dilute aqueous electrolyte has a very high ionic conductivity. The increase of salt concentration accompanies with the decrease of the ionic conductivity from 34.35 ms cm^{-1} to 2.21 ms cm^{-1}, but this value maintained the same magnitude as compared to the conventional organic electrolyte (~ 1 ms cm^{-1}).

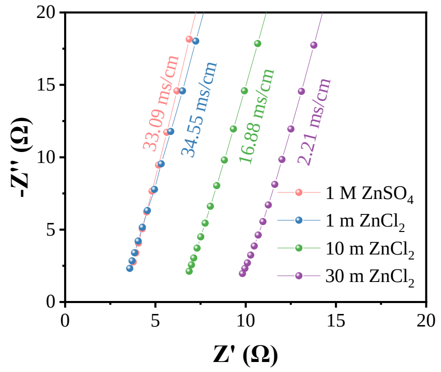

Figure S12 The Nyquist plots of different electrolytes (1 M $ZnSO_4$, 1 m $ZnCl_2$, 10 m $ZnCl_2$ and 30 m $ZnCl_2$).

To clarify the potential of V_2O_5 cathode in WiSE, we synthesized the polyaniline intercalated V_2O_5 nanosheet and test the cycling performance in WiSE. Typically, commercial V_2O_5 (3 g) powder were added into distilled water (500 mL) under vigorous stirring at room temperature. Then, aniline (1 mL) was added into above solution with continuous stirring. Later, the pH value of the precursor solution was adjusted to 3 through the addition of hydrochloric acid (3 mol L^{-1}). Subsequently, the dark green solution was transferred to a 1 L beaker, heated via water bath at 80 °C and held for 24 h, then cooled down to room temperature. The obtained dark green precipitate was collected and washed 3 times with distilled water and anhydrous alcohol, then dried in a vacuum at 60 °C for 24 h and kept for further characterization. The polyaniline intercalated V_2O_5 can deliver a capacity up to 420 mAh g^{-1}, reaching the theoretical value of three electron transfer (440 m Ah g^{-1}).

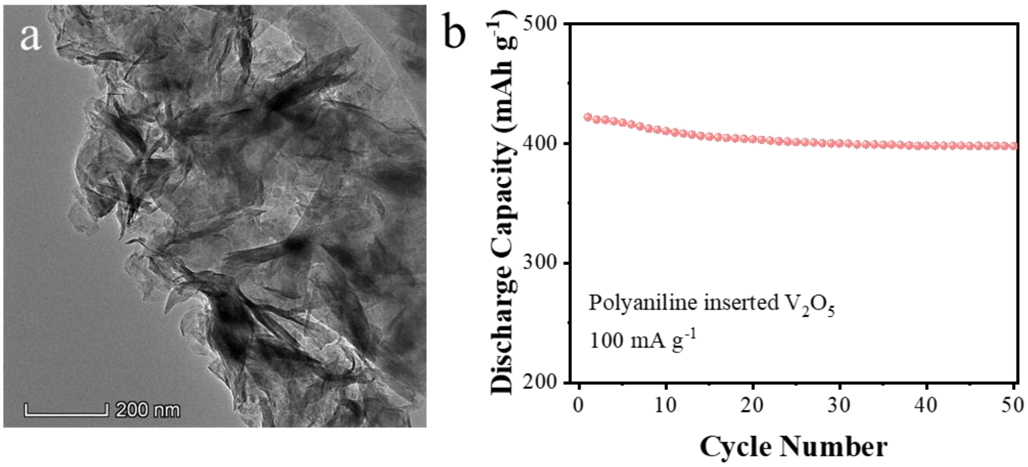

Figure S13 a) The TEM image of polyaniline inserted V_2O_5. b) The cycling performance of polyaniline inserted V_2O_5 in 30 m $ZnCl_2$.

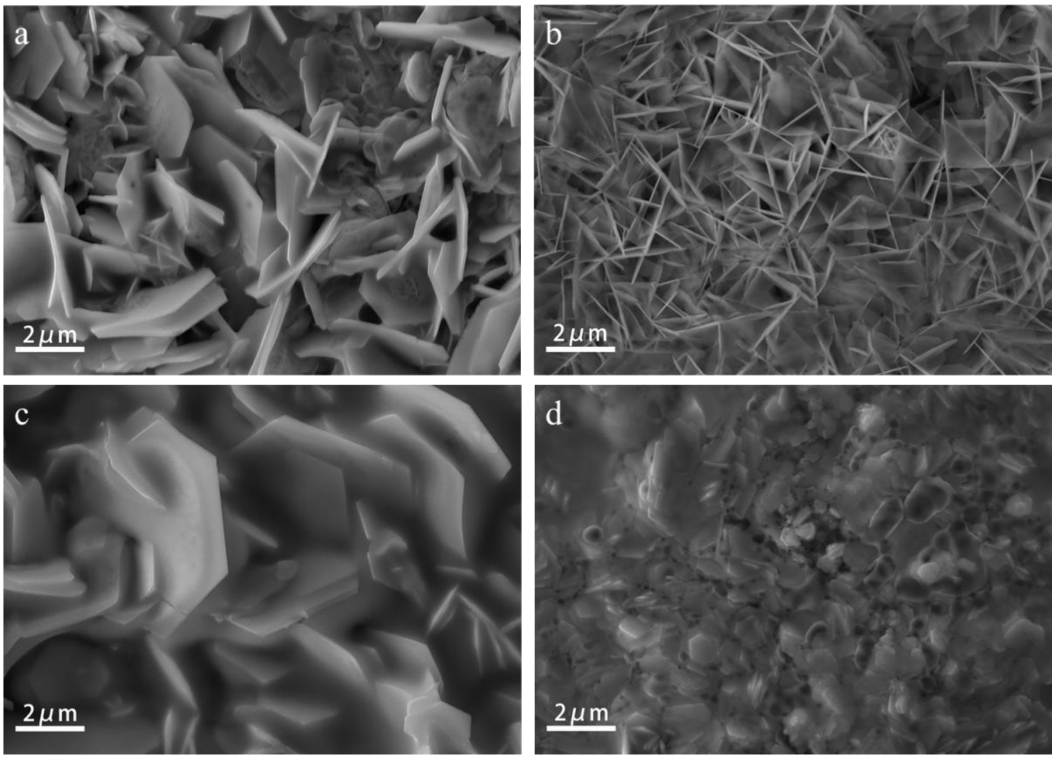

Figure S14 SEM images of the Zn electrodes cycled in a) 1 M $ZnSO_4$, b) 1 m $ZnCl_2$, c) 10 m $ZnCl_2$ and d) 30 m $ZnCl_2$ at 0.1 A g^{-1} for 200 cycles.

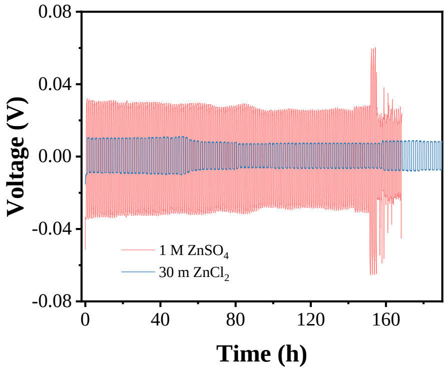

Figure S15 Galvanostatic cycling curves of Zn foil symmetrical cells in different electrolytes at a current density of 2 mA cm^{-2} at an areal capacity of 1 mAh cm^{-2}.

We test the coulombic efficiency of zinc metal to evaluate the side reactions occurred on zinc metal in asymmetric Zn||Zn cells with titanium foil as the current collector. For the repeated Zn plating/stripping tests of asymmetric cells, the capacity of Zn plating was fixed at 1 mAh cm^{-2} under 0.5 mA cm^{-2}, and the cutoff voltage of stripping process was set at 1.0 V. A pre-cycling of cells was carried out for three cycles to stabilize the cell performance. In this asymmetric cell, plating/stripping in 30 m $ZnCl_2$ demonstrates a much higher average CE of 98.4% than 92.3% in 1 M $ZnSO_4$. Therefore, the H_2 evolution induced by the low pH value is not a critical concern in concentrated electrolyte.

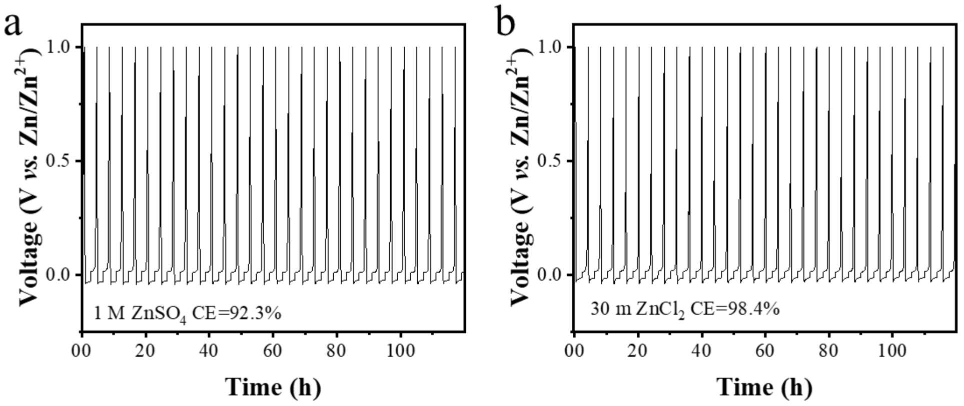

Figure S16 CE measurements of Zn plating/stripping in asymmetric Zn||Zn cells in (a) 1 M $ZnSO_4$ and (b) 30 m $ZnCl_2$ electrolytes.

Sample	Label	R_{ct} [Ω]	-Log_{10} diffusion coefficient [cm^2 s^{-1}]
1 m $ZnSO_4$	1 st	175.4	12.6
	10 th	6.93	12.0
30 m $ZnCl_2$	1 st	13.96	13.4
	10 th	21.86	14.2

Table S1. The charge transfer resistance and ion diffusion coefficients from the EIS tests.
